# Enhancement of Fluoride Retention in Human Enamel Using Low-Energy Blue Diode Laser (445 nm): An Ex Vivo Study

**DOI:** 10.3390/mi16121349

**Published:** 2025-11-28

**Authors:** Melanie Namour, Marwan El Mobadder, Ilaria Giovannacci, Alain Vanheusden, Samir Nammour

**Affiliations:** 1 Department of Dental Science, Faculty of Medicine, University of Liege, 4000 Liege, Belgiumalain.vanheusden@uliege.be (A.V.); 2 Laser Laboratory, Oral Surgery Department, Wroclaw Medical University, 50-425 Wroclaw, Poland; marwan.mobader@gmail.com; 3 Unît of Oral Surgery, Oral Medicine and Laser Therapy, University of Parma, 43121 Parma, Italy

**Keywords:** enamel, human enamel, fluoride, fluoride retention, 445 nm diode laser, fluoridation, ex vivo study, caries prevention, acid resistance

## Abstract

Aim: This ex vivo study aimed to evaluate the effect of low-energy 445 nm diode laser irradiation on permanent fluoride retention in human enamel. Materials and Methods: Eighty caries-free extracted permanent human teeth were used to prepare 480 enamel discs (2 × 2 mm). Baseline fluoride content in untreated enamel specimens (control group E) was measured using particle-induced gamma-ray emission (PIGE). All specimens then received a topical application of acidulated phosphate fluoride for 5 min, followed by rinsing with double-distilled water for 1 min. Fluoride quantification was subsequently repeated. Specimens were randomly allocated into two groups: fluoridated only (EF; n = 240) and fluoridated plus laser-treated (EFL; n = 240). Each group was further subdivided based on storage conditions: either in air or in double-distilled water at 36 °C for 7 days. Laser irradiation was performed using a 445 nm diode laser in continuous-wave mode at 350 mW for 30 s, with a beam diameter of 10 mm, an energy density of 13.375 J/cm^2^, and a power density of 0.445 W/cm^2^. Results: At baseline, mean fluoride content across all specimens was 702.23 ± 201 ppm. Immediately after fluoridation, fluoride levels increased to 11,059 ± 386 ppm in the EF group and 10,842 ± 234 ppm in the EFL group, with no significant difference between groups. After 7 days of storage in air, fluoride retention decreased to 5714 ± 1162 ppm in EF and 5973 ± 861 ppm in EFL, again without significant difference. However, after 7 days of immersion in double-distilled water, the EF group exhibited complete loss of acquired fluoride, with values falling below baseline (337 ± 150 ppm). In contrast, the EFL group retained a substantial portion of the fluoride acquired during fluoridation (total 1533 ± 163 ppm), indicating that laser irradiation significantly prevented fluoride loss (*p* < 0.001). Conclusions: Low-energy 445 nm diode laser irradiation of fluoridated enamel significantly enhances fluoride retention under aqueous conditions simulating osmotic processes. Laser treatment preserved a substantial portion of fluoride acquired during fluoridation, whereas fluoridated but unlased enamel lost nearly all fluoride, with levels dropping below baseline. This approach may offer clinical benefits for improving enamel fluoride enrichment, thereby increasing resistance to acid challenge and reducing caries risk.

## 1. Introduction

Dental caries prevention remains a major objective in restorative dentistry, with various strategies aimed at enhancing enamel resistance and promoting long-term fluoride retention. Traditional approaches primarily involve oral hygiene education and topical fluoride application, which facilitate enamel remineralization and reduce susceptibility to acid dissolution [1,2]. However, the efficacy of topical fluoride is often compromised by its limited retention on the enamel surface, as a substantial proportion is lost due to salivary clearance, mastication, and dietary acids [3,4]. Consequently, improving fluoride incorporation within enamel structures represents a critical challenge in preventive dentistry.

Over the years, laser technology has garnered significant attention as a conservative and non-invasive method for modifying dental hard tissues and enhancing their acid resistance [5,6]. Experimental and clinical studies have demonstrated that specific laser wavelengths, applied under controlled high-energy parameters, can induce morphological and physicochemical changes in enamel that promote increased acid resistance [7,8,9]. These alterations may include modifications to the crystal structure, reduction in carbonate content, surface melting, and fusion of hydroxyapatite crystals, all contributing to decreased solubility and enhanced acid resistance [10,11,12]. Despite these promising findings, the precise mechanisms by which laser irradiation improves enamel acid resistance remain incompletely understood. In particular, the synergistic effects of combining laser irradiation with fluoride application, as well as the influence of different laser wavelengths and energy densities, have yet to be fully elucidated.

Previous studies have reported heterogeneous outcomes regarding the use of high-energy laser irradiation at various wavelengths (CO_2_, Nd:YAG, Er:YAG, argon, and diode lasers) to enhance the acid resistance of dental enamel following topical fluoride application [13,14,15,16,17,18,19]. Moreover, there is a scarcity of research investigating the effects of low-energy 445 nm diode laser irradiation combined with fluoride application to mitigate enamel demineralization after acid challenge [7]. The processes behind laser-induced acid resistance in enamel are not yet fully understood. Fluoride incorporation into the enamel structure may play a key role, and studies using precise analytical methods to measure fluoride retention after combined laser and fluoride application could offer valuable insight into this process.

Despite promising results with other wavelengths, the effect of the 445 nm diode laser, a relatively new device in dentistry, on enamel fluoride uptake remains largely unexplored. Additionally, the potential for low-energy 445 nm diode laser irradiation to enhance fluoride retention following topical application carries important clinical implications. If proven effective, this approach could serve as a minimally invasive method to extend the cariostatic benefits of fluoride and protect enamel against demineralization. Fluoride accumulation in enamel is known to strengthen resistance to acid attack and caries, highlighting the importance of investigating techniques that promote its long-term retention.

Therefore, the aim of this study is to evaluate whether 445 nm diode laser irradiation, in combination with topical fluoride application, can enhance fluoride retention in human enamel. Fluoride uptake was quantified using particle-induced gamma-ray emission (PIGE), a highly sensitive and precise method for elemental analysis.

The null hypothesis posited no significant difference in fluoride retention in dental enamel between samples treated with topical fluoride alone and those treated with topical fluoride combined with 445 nm diode laser irradiation.

## 2. Materials and Methods

### 2.1. Study Design and Sample Preparation

This in vitro study aimed to investigate the effect of 445 nm diode laser irradiation at low energy density on fluoride retention in enamel. A total of 80 freshly extracted, caries-free human permanent teeth were collected and stored in 0.1% thymol solution at 4 °C until use. The solution was replaced weekly to maintain the structural integrity of the enamel.

Informed consent was obtained from all patients, authorizing the use of their extracted teeth for research purposes. The collected teeth had been extracted for various reasons unrelated to this study and were unknown to the authors. As the research did not involve patients or animals, prior approval from the university ethics committee was not required.

**Figure 1:**
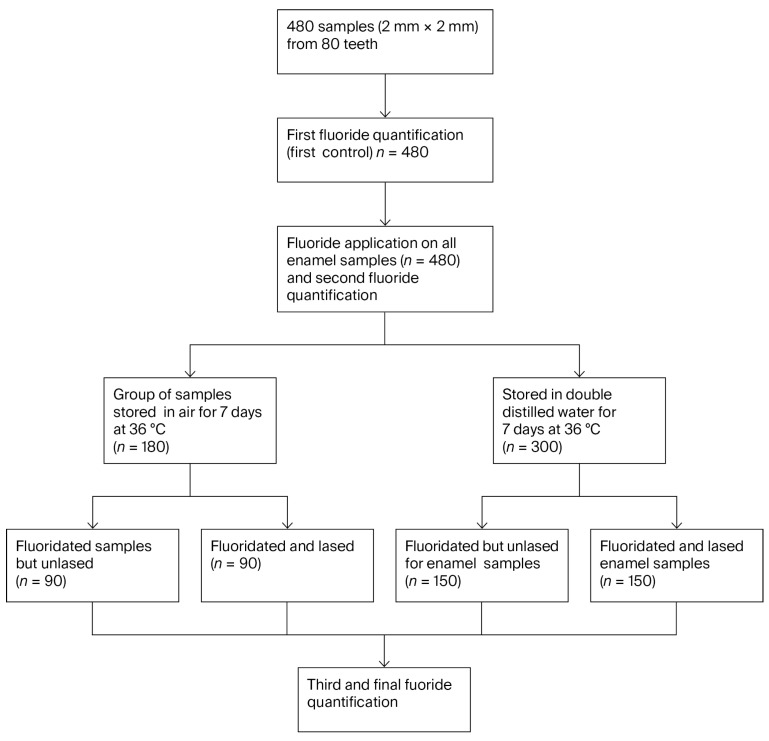
Schematic illustration of the study design, including specimen preparation, fluoride and laser treatments, allocation into treatment groups (EF, EFL) and storage conditions (air or double-distilled water at 36 °C).

A total of 480 enamel samples (2 × 2 mm discs) were prepared from 80 extracted human teeth. The fluoride content of all samples was first quantified to establish baseline values (first control). Subsequently, all samples underwent topical fluoride application, followed by a second fluoride quantification (second control). The samples were then randomly allocated into two main groups: a fluoridated but non-laser-treated group (EF, n = 240) and a fluoridated and laser-treated group (EFL, n = 240). Each group was further subdivided according to storage conditions: samples stored in air for 7 days (EF-Air, n = 90; EFL-Air, n = 90) and samples stored in double-distilled water for 7 days (EF-Water, n = 150; EFL-Water, n = 150) (Figure 1). The distilled water was changed daily to ensure optimal simulation of osmotic processes on the enamel surfaces. This experimental design allowed evaluation of the effect of laser treatment on fluoride retention in enamel under different storage conditions. The protocols for laser irradiation and fluoride quantification are described in the following sections.

**Figure 2:**
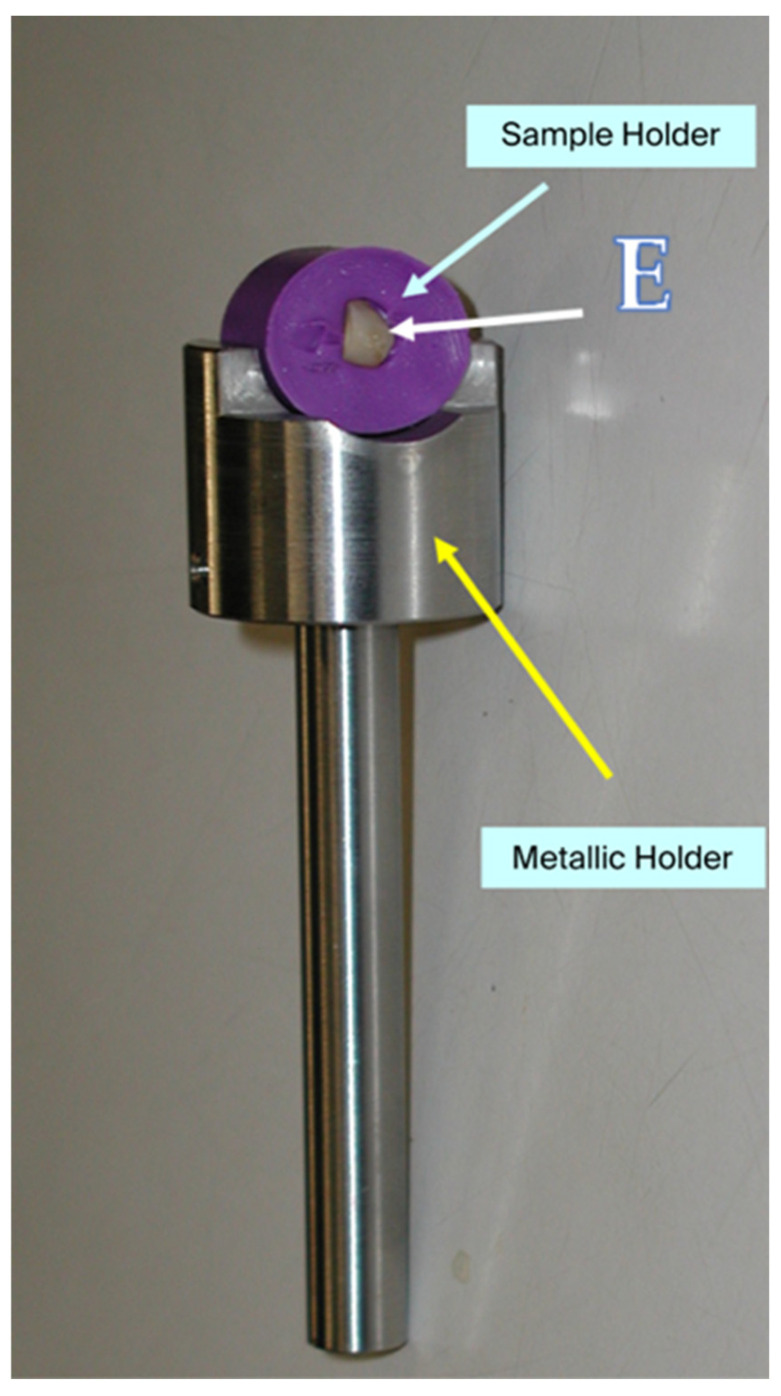
Enamel specimen (E) positioned in a fixed target holder designed to ensure consistent analysis of the same 1 mm^2^ area before and after all treatments and controls.

**Figure 3:**
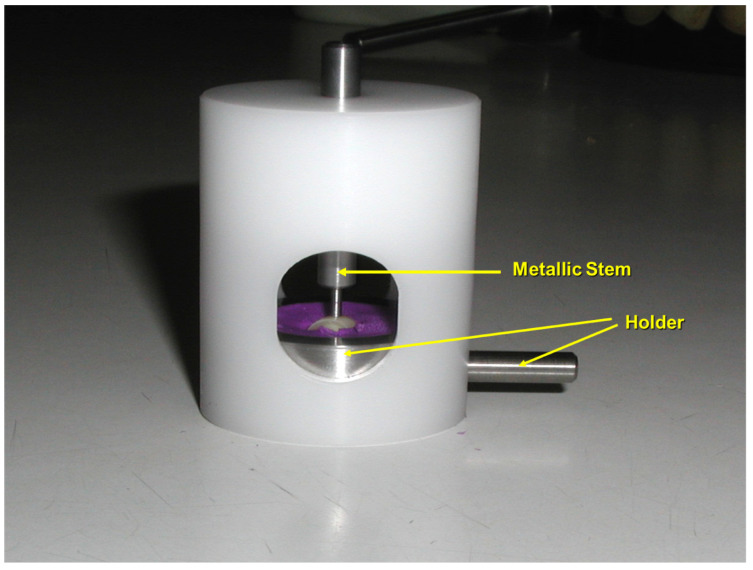
Simulation of the proton beam trajectory (indicated by the metallic stem) and verification of proper enamel specimen positioning within the holder prior to PIGE measurements.

Because the baseline fluoride distribution on the enamel surface is not homogeneous, particular care was taken to evaluate the same enamel area at each experimental step. To achieve this, a custom-designed sample holder was manufactured, allowing precise repositioning of each specimen and ensuring that repeated analyses were performed on the same 1 mm^2^ area of enamel surface for every sample (Figure 2 and Figure 3).

### 2.2. Fluoride Application

Topical fluoridation was performed using an acidulated phosphate fluoride (APF) foam (Oral-B Minute-Foam; Procter & Gamble, Cincinnati, OH, USA) containing 1.23% fluoride ion (*w*/*w*). The foam was applied uniformly to the enamel surfaces of all specimens for 5 min, followed by a 1 min rinse with double-distilled water to remove excess fluoride.

### 2.3. Laser Irradiation

A diode laser with a wavelength of 445 nm (Yuwei Dental Laser, Yuwei Photoelectric Technology, Beijing, China) was used for enamel irradiation. A laboratory modification was implemented to expand the beam diameter to 10 mm at the target surface. The actual delivered power was verified using a calibrated laser power meter (Gentec Electro-Optics, Quebec, QC, Canada). The laser operated in continuous-wave mode with an output power of 350 mW. Irradiation was applied for 30 s with a 10 mm beam diameter, corresponding to an energy density of 13.375 J/cm^2^ and a power density of 0.445 W/cm^2^.

### 2.4. Fluoride Quantification by PIGE

**Figure 4:**
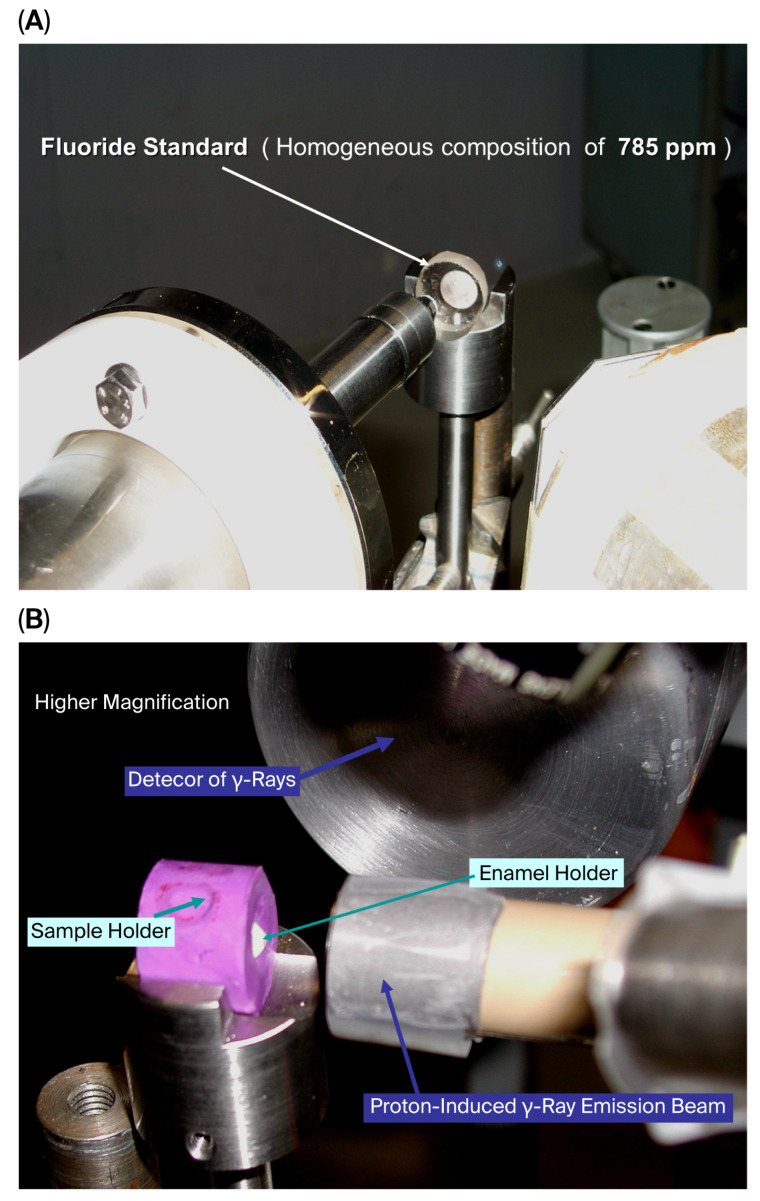
(**A**) Enamel specimen positioned within the fixed target holder, shown alongside the proton beam and gamma-ray detector of the Particle-Induced Gamma-ray Emission (PIGE) system. (**B**) Fluoride standard used for calibration of fluoride quantification, positioned within the fixed target holder next to the gamma-ray detector of the PIGE system.

The fluoride content of enamel was quantitatively assessed using Proton-Induced Gamma-ray Emission (PIGE), following established protocols [10,20]. A 3.2 MeV proton beam, generated by a Tandetron accelerator, was directed onto a thin tantalum foil before penetrating the superficial 20 µm of enamel. Irradiation was performed outside the accelerator vacuum, and prompt γ-rays emitted by light and medium elements were detected using an energy-dispersive detector (Figure 4). Measurement reproducibility was monitored through tantalum γ-ray signals. Each analysis was conducted for 30 s with a beam intensity of 30 nA, probing an enamel surface area of 1 mm^2^ to a depth of approximately 20 µm. The measurement error was approximately 1%. Sequential PIGE analyses were performed at baseline and after fluoride application for all samples, and subsequently for all experimental groups.

### 2.5. Statistical Analysis

Statistical analyses were performed using GraphPad Prism version 10.0 (GraphPad Software, Inc., La Jolla, CA, USA). Fluoride retention values were expressed in parts per million (ppm) and reported as means ± standard deviations. Data normality was assessed using the D’Agostino–Pearson omnibus test. Intergroup comparisons were conducted using repeated-measures analysis of variance (ANOVA), followed by Newman–Keuls post hoc tests. A *p*-value of <0.05 was considered statistically significant.

## 3. Results

### 3.1. Fluoride Adsorption Immediately After Fluoridation

At baseline, prior to fluoride application, the mean fluoride retention across all enamel specimens was 702.23 ± 201 ppm (n = 480). Immediately following fluoride application, the mean fluoride retentions in the fluoride-treated group (EF) and the fluoride-treated plus laser-irradiated group (EFL) were 11,059 ± 386 ppm and 10,842.27 ± 234 ppm, respectively (Table 1). These values confirm a substantial increase in fluoride uptake after topical application compared to baseline. No statistically significant difference was observed between the EF and EFL groups on day 0 (prior to any further experimentation).

### 3.2. Fluoride Retention After 7 Days in Air

After 7 days of storage in air at 36 °C, the fluoride-treated group (EF) exhibited a mean fluoride retention of 5714.34 ± 3162 ppm. In comparison, the fluoride-treated and laser-irradiated group (EFL) showed a slightly higher mean value of 5972.98 ± 3061 ppm (Table 2). Repeated measures ANOVA (95% confidence interval; *p* > 0.05) indicated that the difference in fluoride retention between the EF and EFL groups was not statistically significant under these conditions.

### 3.3. Fluoride Retention After 7 Days of Immersion in Distilled Water

After 7 days of immersion in double-distilled water at 36 °C, the fluoride-treated group (EF) exhibited a dramatic reduction in fluoride retention, with a mean value of 337.41 ± 149.57 ppm, which was significantly lower than its initial level on Day 0 (11,059.44 ± 386 ppm, *p* < 0.001). This decrease was so pronounced that the EF group not only lost all fluoride acquired during the fluoridation process but also fell below the baseline level (702.23 ± 201 ppm, *p* < 0.001).

**Figure 5:**
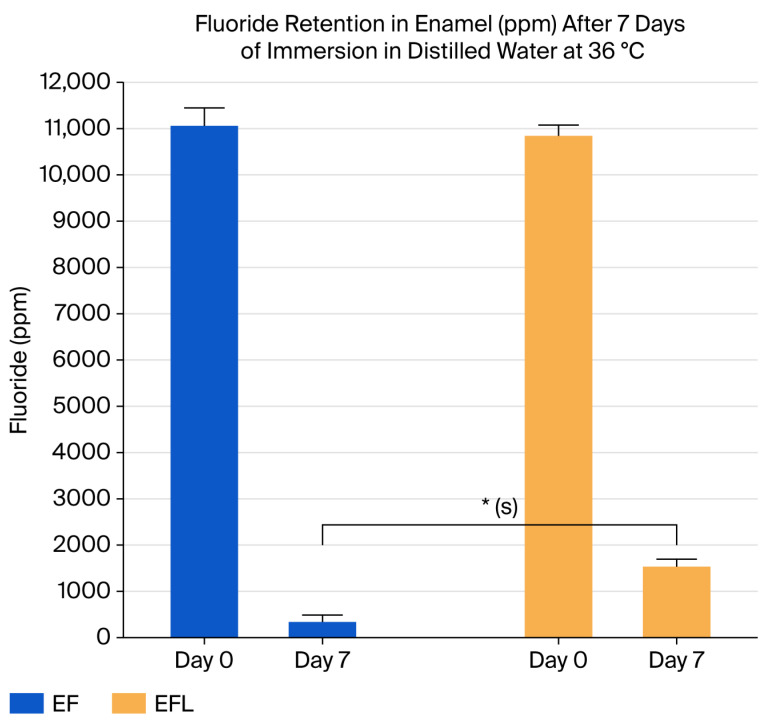
Illustration of the comparative fluoride retention after 7 days of immersion in double-distilled water. The EF group showed a drastic reduction to approximately 337 ppm, whereas the EFL group retained significantly higher levels at around 1533 ppm, highlighting the protective effect of laser irradiation (*p* < 0.001). * highly significant difference between EF and EFL after 7 days (*p* < 0.001).

In contrast, the fluoride-treated and laser-irradiated group (EFL) demonstrated a markedly better performance, maintaining a mean fluoride retention of 1533.11 ± 162 ppm after 7 days. Although this value was significantly lower than its Day 0 level (10,842.27 ± 234 ppm, *p* < 0.001), it remained substantially higher than the EF group after the same period (*p* < 0.001) (Table 3 and Figure 5).

Overall, statistical analysis using one-way ANOVA with Bartlett’s correction confirmed a highly significant difference between EF and EFL after 7 days (*p* < 0.001), leading to rejection of the null hypothesis. These findings indicate that laser irradiation plays a critical role in preserving fluoride on enamel surfaces under prolonged water immersion, simulating an osmosis-like process.

**Figure 6:**
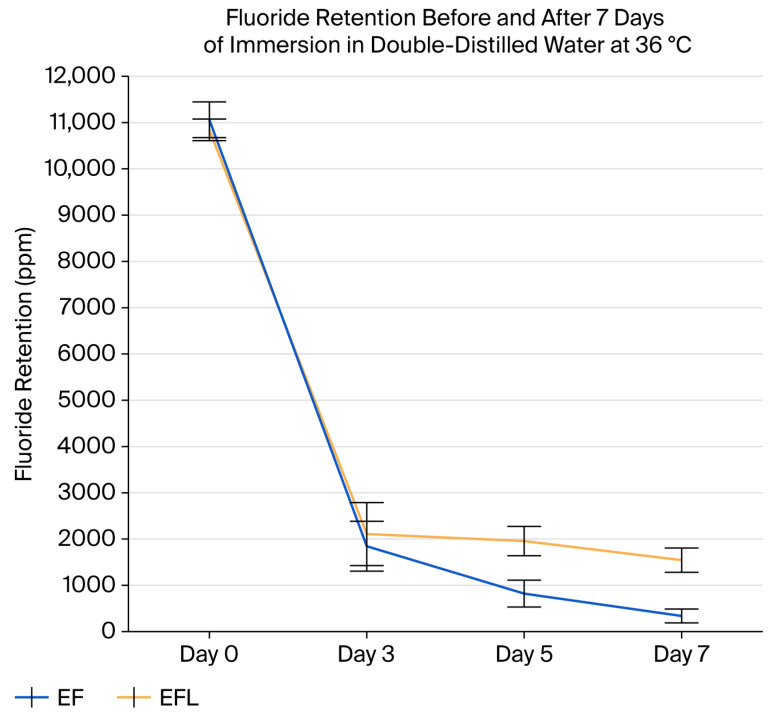
Fluoride retention in enamel on days 3, 5, and 7 during immersion in double-distilled water at 36 °C. By day 7, the fluoride-treated group (EF; fluoridated samples only) exhibited a significant reduction to approximately 337 ± 150 ppm, whereas the fluoride-treated and laser-irradiated group (EFL; fluoridated and lased samples) retained substantially higher levels at about 1533 ± 163 ppm.

Figure 6 illustrates the progressive reduction in fluoride retention over time (Days 3, 5, and 7) for both groups. The EF group showed a steep and continuous decline, reaching near-complete fluoride loss by Day 7, whereas the EFL group exhibited a slower rate of reduction, maintaining considerably higher fluoride levels throughout the observation period.

## 4. Discussion

The present ex vivo study demonstrates that low-energy 445 nm diode laser irradiation applied after topical fluoride treatment significantly enhances fluoride retention on enamel surfaces. While fluoridated but unlased enamel (group EF) lost nearly all its adsorbed fluoride after 7 days of immersion in double-distilled water, laser-treated enamel (group EFL) preserved a substantial fraction—approximately 14% (1533.11 ppm) of the initially retained fluoride (10,842.27 ppm). These findings suggest that 445 nm diode laser irradiation, under the parameters used (350 mW, 30 s, 13.375 J/cm^2^, and 0.445 W/cm^2^), can modify enamel structure in a way that stabilizes fluoride retention and potentially prolongs its cariostatic effect.

Fluoride remains a cornerstone in caries prevention by reducing enamel solubility under acid challenge [10,19,20,21,22,23]. Topical fluoride applications create a surface reservoir that gradually releases fluoride, providing sustained protection against demineralization [1,3,4]. The ability to maintain higher fluoride levels on enamel surfaces is therefore of considerable clinical importance. Our data indicate that 445 nm diode laser irradiation enhances this fluoride reservoir, particularly under severe conditions simulating osmotic-like processes rather than an oral environment, where saliva and other fluids would otherwise remove surface-bound fluoride.

The literature reports numerous studies investigating low-energy laser irradiation across a wide range of wavelengths (CO_2_, Nd:YAG, Er:YAG, argon, and diode lasers) [7,11,12,17,18,19,21,22,23,24,25,26,27,28,29] in combination with fluoride to mitigate enamel demineralization following acid challenge. For example, low-energy Er:YAG lasers (wavelength: 2.94 μm; fluence: 8 J/cm^2^) combined with fluoride varnish have demonstrated improved microhardness and reduced mineral loss in primary enamel, with treated surfaces showing up to 25% higher resistance to acid attacks compared to fluoride alone in pH-cycling models [22]. Similarly, diode lasers at 810 nm (power: 1 W, energy density: 30 J/cm^2^) used with topical fluoride have been shown to enhance enamel remineralization in deciduous teeth, resulting in a 40% reduction in erosion depth during acid challenges versus untreated controls [23].

Low-energy CO_2_ lasers (wavelength: 10.6 μm; fluence: 10 J/cm^2^) applied after amine fluoride treatment have increased fluoride retention and acid resistance, with ex vivo studies reporting 50% less demineralization in laser-fluoride groups compared to fluoride-only applications [21]. Argon lasers (wavelength: 488–514 nm; power: 250 mW, fluence: 12 J/cm^2^) combined with APF have also reduced enamel decalcification by 60% in orthodontic models, an effect attributed to enhanced fluoride incorporation without thermal damage [19,26].

The mechanisms underlying the increase in enamel acid resistance remain incompletely understood, underscoring the need for further clarification. This consideration motivated our study, which employed precise analytical techniques to quantify fluoride retention in enamel following combined laser-fluoride protocols. Our findings suggest that the observed enhancement of enamel acid resistance may be attributed to increased fluoride retention or incorporation into the enamel structure.

Additionally, these results hold considerable clinical relevance, as they may offer a minimally invasive strategy to enrich dental enamel with fluoride, aiming to prolong its cariostatic effect and mitigate enamel demineralization. An increase in enamel fluoride concentration could contribute to greater protection against demineralization and caries development.

The enhancement of fluoride stabilization on fluoridated and laser-irradiated enamel at low energy may be attributable to the interaction between visible laser light (445 nm) and enamel hydroxyapatite (HAP) in the presence of fluoride. Given the absorption capacity of visible laser light by atomic electrons [30], it is plausible that the energy of the laser could be absorbed by OH^−^ ions within HAP, leading to transient disruption or weakening of their ionic bonds. In the presence of fluoride ions, the most electronegative element in the periodic table, these ions may preferentially bond to HAP, replacing OH^−^ groups. Consequently, the modified HAP structure enriched with fluoride could transform into fluorapatite (FAP), thereby explaining the observed enhancement of fluoride retention in fluoridated and laser-irradiated enamel.

Clinically, the combination of topical fluoride and low-energy diode laser irradiation represents a minimally invasive and easily implemented strategy to reinforce caries prevention. This approach may be particularly valuable for patients at elevated caries risk, including those with xerostomia, orthodontic appliances, or high dietary sugar exposure.

Importantly, previous studies have reported that low-energy laser irradiation, similar to the parameters used in our study, produces negligible thermal effects, which are considered harmless for pulp vitality [31,32]. Our findings also highlight a critical limitation of standard topical fluoridation: fluoride adsorbed without laser irradiation is highly susceptible to removal in aqueous environments through osmosis-like processes, as demonstrated by the complete loss of surface fluoride in the fluoridated-only group after immersion.

Several limitations must be acknowledged. This investigation was conducted in vitro under controlled static conditions that only partially replicate the oral environment, including salivary flow, biofilm activity, and mechanical forces. Additionally, PIGE analysis, while highly precise for quantifying fluoride in the superficial 20 µm of enamel, does not assess deeper incorporation or long-term fluoride release kinetics under physiological conditions. Consequently, extrapolation of these results to in vivo efficacy should be approached with caution.

Nevertheless, laser treatment appears to mitigate fluoride loss, suggesting potential for longer-lasting fluoride efficacy. However, the precise physicochemical interactions responsible for this effect, whether enhanced surface adsorption, diffusion into enamel microstructure, or formation of novel fluoride-calcium phases, remain unclear and warrant further mechanistic investigation.

Topical fluoride retention varies according to product type, application method, and oral conditions, with fluoride primarily forming CaF_2_-like deposits on enamel that act as reservoirs for gradual release [1,2,3]. Acidulated phosphate fluoride (APF) foam (1.23% F) exhibits lower initial retention than gels due to its smaller material volume and lower density, resulting in faster oral clearance but reduced systemic exposure, an advantage in pediatric patients [1,2]. Immediately after application, foam produces measurable increases in salivary and enamel fluoride; however, these levels decline to baseline within approximately two weeks, reflecting the gradual dissolution of CaF_2_ deposits [1,2,3]. Despite this transient retention, protective effects may persist through fluorapatite formation, supporting reapplication every 3–6 months in high-risk patients [33,34,35].

In the present study, enamel samples were immersed in double-distilled water to simulate osmotic processes occurring in the oral cavity. This approach was intended to accelerate the release of loosely adsorbed fluoride while preserving the fraction permanently incorporated into the enamel lattice. By creating an osmosis-like environment, we reduced the total monitoring time required for the experiment while mimicking long-term in vivo fluoride dynamics. This method allowed accurate distinction and quantification of enamel fluoride uptake and retention, providing a reliable in vitro model to evaluate the efficacy of topical fluoride treatments under conditions approximating the dynamic oral environment.

Additionally, fluoride concentration in enamel is a critical factor in enhancing acid resistance and reducing caries incidence. Studies indicate that even low fluoride exposures can inhibit demineralization of sound enamel, while higher concentrations are needed to promote remineralization of early lesions [36]. In situ and in vitro experiments suggest that enamel exposed to fluoride concentrations above approximately 1000 ppm demonstrates significantly improved resistance to acid challenges and cariogenic attacks [37,38]. Topical fluoride applications delivering concentrations greater than 1000 ppm, such as standard APF gels and foams, have been associated with measurable increases in enamel fluoride content and long-term cariostatic effects [38,39]. In the present study, laser irradiation of fluoridated enamel resulted in high, permanent fluoride retention, with 1533 ppm incorporated into the enamel structure, suggesting that treated surfaces are likely to exhibit enhanced acid resistance and reduced susceptibility to caries development under normal oral conditions.

Future research should focus on in vivo validation of laser-assisted fluoridation, assessing its remineralization potential and caries-preventive efficacy under dynamic oral conditions. Mechanistic studies are needed to clarify the microstructural and chemical changes induced by 445 nm diode laser irradiation and their role in fluoride stabilization. Optimization of laser parameters—including fluence, wavelength, and exposure time—will be critical to maximize fluoride stabilization while ensuring pulp safety.

Furthermore, the use of biocompatible fluoride products remains an essential consideration. Several formulations are available for topical application on enamel and dentin, but ensuring their non-toxicity and compatibility with dental tissues is vital to prevent potential pulp damage. Recently, López García et al. [40] demonstrated that ammonia-free silver fluoride formulations are biocompatible and could be considered for future studies targeting fluoride enrichment of dentin in vital teeth.

Our study may represent a promising adjunctive strategy in preventive dentistry, offering a minimally invasive and clinically feasible method to prolong the protective effects of fluoride. These findings support further in vivo and mechanistic investigations to confirm efficacy and elucidate the underlying processes.

## 5. Conclusions

This study demonstrates that low-energy 445 nm diode laser irradiation, applied under controlled parameters and low energy density, significantly enhances fluoride stabilization and retention in human enamel under osmosis-like conditions. These findings suggest that laser-assisted fluoridation may represent a clinically promising approach to improve enamel fluoride enrichment, thereby increasing resistance to acid challenge and reducing the risk of caries formation.

## Figures and Tables

**Table 1:** Means and standard deviations of fluoride retention on enamel surfaces before and after fluoride application. Values are expressed as mean ± standard deviation. *p* < 0.05 was considered statistically significant. * Significant difference compared to baseline; ^ns^ = non-significant difference.

	Group E Baseline (n = 480)	After Fluoride Application Day 0 (EF, n = 240)	Fluoride Application + Laser Irradiation (Day 0) (EFL, n = 240)
**Mean (in PPM)**	702.23	11,059.44 *	10,842.27 * ^ns^
**Standard deviation**	201	386	234

**Table 2:** Means and standard deviations of fluoride retention on enamel surfaces before and after 7 days of storage in air. Values are expressed as mean ± standard deviation. *p* < 0.05 was considered statistically significant. * Significant difference compared to baseline; ** Significant difference compared to Day 0 within the same group; ^ns^ = non-significant difference between EF and EFL after 7 days.

	E (Control at Baseline)	EF Day 0	EF Day 7	EFL Day 0	EFL Day 7
Mean (ppm)	702.23	11,059.44 *	5714.61 **	10,842.27 *	5973.84 ** ^ns^
Std. Deviation	201	386	3162	234	3061

**Table 3:** Means and standard deviations of fluoride retention on enamel surfaces after 7 days of immersion in double-distilled water. Values are expressed as mean ± standard deviation. *p* < 0.05 was considered statistically significant. * Significant difference compared to baseline; ** Significant difference compared to Day 0 within the same group; *** highly significant difference between EF and EFL after 7 days (*p* < 0.001).

	E (at Baseline (n = 480)	EF Day 0 (n = 150)	EF Day 7 (n = 150)	EFL Day 0 (n = 150)	EFL Day 7 (n = 150)
Mean value (ppm)	702.23	11,059.44 *	337.41 **	10,842.27 *	1533.11 ***
Standard deviation	201	386	149.57	234	162.81

## Data Availability

The original contributions presented in this study are included in the article. Further inquiries can be directed to the corresponding author.
